# Target animal safety evaluation of a novel topical combination of esafoxolaner, eprinomectin and praziquantel for cats

**DOI:** 10.1051/parasite/2021015

**Published:** 2021-04-02

**Authors:** Aradhana Gupta, Christine Baker, Hailun Wang, Norba Targa, Anthony Pfefferkorn, Eric Tielemans

**Affiliations:** 1 Boehringer-Ingelheim Animal Health 3239 Satellite Blvd Duluth 30096 GA USA (until 2020); 2 Boehringer-Ingelheim Animal Health 1730 Olympic Drive Athens 30601 GA USA; 3 Boehringer-Ingelheim Animal Health 29 Avenue Tony Garnier 69007 Lyon France

**Keywords:** Cat, Esafoxolaner, Eprinomectin, Praziquantel, Safety, Topical

## Abstract

The safety profile of NexGard^®^ Combo, a novel topical product for cats combining esafoxolaner, eprinomectin and praziquantel, for the treatment and prevention of internal and external parasites, was evaluated in kittens, in two margin-of-safety studies (Studies #1 and #2), and in an oral tolerance study (Study #3). In the margin of safety studies, kittens were dosed several times topically with multiples of the maximum exposure dose (1×): in Study #1, 3× and 5× doses four times at 2-week intervals; in Study #2, 1×, 3× and 5× doses six times at 4-week intervals. In Study #3, kittens were dosed orally once with a 1× dose. Furthermore, in Study #1, another group of kittens was dosed topically twice at a 4-week interval with a formulation of esafoxolaner as the sole active ingredient dosed at 23×. Physical examinations and clinical pathology analyses were performed throughout the studies, followed by necropsy and detailed histopathological evaluation in Studies #1 and #2. No significant treatment related effects were observed in the three studies, except for one occurrence of reversible neurological signs attributed to eprinomectin in one cat after the third 5× dose in Study #2, with clinical signs observed nine hours after dosing, pronounced for a few hours, significantly improved the next day, and absent 2 days after dosing. In conclusion, NexGard^®^ Combo was demonstrated safe in kittens following repeated topical administrations and following oral ingestion, and very high topical doses of esafoxolaner were well tolerated.

## Abstract (fr)

Le profil de sécurité de NexGard^®^ Combo, un nouveau produit topique destiné aux chats associant l’esafoxolaner, l’éprinomectine et le praziquantel, pour le traitement et la prévention des parasites internes et externes, a été évalué chez les chatons, dans deux études de marge de sécurité (études n° 1 et n° 2) et dans une étude de tolérance orale (étude n° 3). Dans les études de marge de sécurité, les chatons ont reçu plusieurs doses topiques avec des multiples de la dose maximale d’exposition (1×) : dans l’étude n° 1, des doses 3× et 5×, quatre fois, à des intervalles de 2 semaines ; dans l’étude n° 2, des doses 1×, 3× et 5×, six fois, à des intervalles de 4 semaines. Dans l’étude n° 3, les chatons ont reçu une dose orale une fois avec une dose 1×. De plus, dans l’étude n° 1, un autre groupe de chatons a reçu une dose topique deux fois à 4 semaines d’intervalle avec une formulation d’esafoxolaner comme seul ingrédient actif dosé à 23×. Des examens physiques et des analyses de pathologie clinique ont été effectués tout au long des études, suivis d’une autopsie et d’une évaluation histopathologique détaillée dans les études n° 1 et n° 2. Aucun effet significatif lié au traitement n’a été observé dans les trois études, à l’exception d’une occurrence de signes neurologiques réversibles attribués à l’éprinomectine chez un chat après la troisième dose 5× dans l’étude n° 2, avec des signes cliniques observés neuf heures après l’administration, prononcés pour quelques heures, considérablement améliorée le lendemain et absent 2 jours après l’administration. En conclusion, NexGard^®^ Combo s’est avéré sûr chez les chatons après des administrations topiques répétées et après une ingestion orale, et des doses topiques très élevées d’esafoxolaner ont été bien tolérées.

## Introduction

NexGard^®^ Combo is a novel combination of three active ingredients, esafoxolaner, eprinomectin, and praziquantel, formulated as a topical solution for cats for the control of infestations by fleas, ticks and mites, the treatment of infections by roundworms, hookworms, and tapeworms, and the prevention of heartworm disease. Esafoxolaner, a novel compound from the isoxazoline class, is the purified and active (S)-enantiomer of afoxolaner, a racemic compound available in oral acaricide and insecticide products for dogs (NexGard^®^ and NexGard SPECTRA^®^), which specifically blocks arthropods ligand-gated chloride ion channels [16]. Eprinomectin, a compound used in topical parasiticide products for cattle [14] and cats [1, 15], is an avermectin of the macrocyclic lactone class [10], active against nematodes. Praziquantel, a well-known compound in veterinary and human medicine, is a pyrazino-isoquinoline derivative anthelminthic that acts specifically on cestode and trematode cell membranes and has been administered safely in oral and transdermal forms for decades [3, 6, 7, 19]. Pharmacokinetic studies showed no interaction in absorption, distribution, metabolism, excretion or efficacy for these three active ingredients [8, 9].

The assessment of the safety of a finished product in the target species is a prerequisite for the registration of veterinary products. The International Cooperation on Harmonisation of Technical Requirements for Registration of Veterinary Medicinal Products (VICH) has developed a guideline (VICH GL 43) for target animal safety studies that requires the compound to be tested using the final commercial formulation at the maximum exposure dose level (1×), and two multiples of this dose (generally three times (3×) and five times (5×)), applied several times in accordance with the recommended duration of use [17]. Safety endpoints are evaluated for the target species at the minimum age defined for product use. Topical antiparasitic products are usually packaged to allow the administration of one package size (one size applicator) to the animal within a specified body weight range. The doses administered to the heaviest animal and the lightest animal in the specified body weight range are designated as the minimum therapeutic dose and the maximum exposure dose, respectively. Each topical dose in a 1×, 3×, and 5× target animal safety study provides 1, 3, and 5 times the maximum exposure dose.

The safety of elevated topical doses of the combination product was evaluated in a preliminary and a regulatory margin-of-safety target animal safety studies, in order to verify whether adverse events may be expected to occur in NexGard^®^ Combo-treated cats. The product may be recommended for monthly administrations and therefore, in the regulatory study, six monthly elevated doses were used to demonstrate the effect of repeated doses. In the preliminary study, four fortnightly doses were used to evaluate the effect of repeated elevated doses applied at shorter frequency; furthermore, esafoxolaner administered as a single compound was specifically evaluated at a very high dose to gain further knowledge about this compound, novel in feline and veterinary medicine. Additionally, safety following oral administration of the topical formulation was evaluated in the target species in a regulatory study, to identify potential effects of accidental ingestion (e.g., by grooming). The three studies were performed in 7–10-week-old kittens.

This article describes the three studies performed for the assessment of the safety profile of NexGard^®^ Combo in the target species.

## Materials and methods

### Ethics

All animal procedures in these studies were reviewed and approved by the Sponsor’s and CRO’s (when applicable) Institutional Animal Care and Use committees, and were in compliance with all applicable sections of the Final Rules of the Animal Welfare Act regulations (9 CFR), the current AVMA Guidelines, and ETS 123 (European Convention for the Protection of Vertebrate Animals used for Experimental and Other Scientific Purposes).

### Test articles

NexGard^®^ Combo combines 1.2% w/v esafoxolaner, 8.3% w/v praziquantel and 0.4% w/v eprinomectin, and is contained in applicators of 0.3 mL or 0.9 mL for topical administration to 0.8 – <2.5 kg (1.8–5.5 lbs) or 2.5–7.5 kg (5.6–16.5 lbs) cats, respectively. The therapeutic dose ranges (minimum–maximum label dose) of esafoxolaner, praziquantel, and eprinomectin in the novel combination product are 1.4–4.5 mg/kg, 10–31 mg/kg and 0.5–1.5 mg/kg, respectively. The 1×, 3× and 5× dose groups (as applicable) were respectively dosed esafoxolaner, praziquantel, and eprinomectin at 4.5, 31.1 and 1.5 mg/kg (1× – 0.375 mL/kg); 13.5, 93.4, and 4.5 mg/kg (3× – 1.125 mL/kg); and 22.5, 155.6, and 7.5 mg/kg (5× – 1.875 mL/kg).

Esafoxolaner used as a single active ingredient in the preliminary study was formulated at 5.5% w/v and was applied topically at 1.875 mL/kg, providing a dose of 103.1 mg/kg, corresponding to 23× the maximum exposure dose provided with NexGard^®^ Combo.

### Study designs

The regulatory studies were designed according to the recommendations of VICH GL 43 [18], and were conducted in accordance with the Food and Drug Administration “Good Laboratory Practice (GLP) Regulations for Non-Clinical Laboratory Studies, 21 CFR Part 58”, which is also accepted under the Organization for Economic Co-operation Development (OECD) Principles of Good Laboratory Practice (Revised 1997, issued January 1998) ENV/MC/CHEM(98)17, OECD Commission Directive 1999/11/EC of March 08, 1999. The preliminary study was designed in accordance with a majority of the principles of VICH GL 43 and was conducted in respect of good scientific practices.

### Study #1: Preliminary margin of safety

The design of Study #1 is summarized in Table 1.

**Table 1:** Study designs.

Target animal safety studies	Preliminary margin-of-safety topical (Study #1)	Regulatory margin-of-safety topical (Study #2)	Regulatory oral tolerance (Study #3)
Investigated product(s)	Esafoxolaner 1.2% w/v, eprinomectin 0.4% w/v, praziquantel 8.3% w/v	NexGard^®^ Combo:	
	Esafoxolaner 5.5% w/v	esafoxolaner 1.2% w/v, eprinomectin 0.4% w/v, praziquantel 8.3% w/v	
Study groups (number of male and female cats)	One control group (no treatment) (1 M, 1 F)	One control group (mineral oil) (4 M, 4 F)	One control group (sterile water) (4 M, 4 F)
	Three treatment groups:	Three treatment groups:	One treatment group:
	3× (3 M, 3 F)	1× NexGard^®^ Combo (4 M, 4 F)	1× NexGard^®^ Combo (4 M, 4 F)
	5× (3M, 4F)	3× NexGard^®^ Combo (4M, 4F)	
	Esafoxolaner 103.1 mg/kg (1.875 mL/kg) (4M, 2F)	5× NexGard^®^ Combo (4M, 4F)	
Number and frequency of treatments	3× and 5× groups: Four topical applications at 2-week intervals (i.e., Days 0, 14, 28 and 42)	Control and all treatment groups: Six topical applications at 4 week intervals (i.e., Days 0, 28, 56, 84, 112 and 140)	Control and the treatment group: single oral administration on Day 0
	Esafoxolaner group: Two topical applications at 4-week intervals (i.e., Days 0 and 28)		
Study variables	Clinical reactions, bodyweight, clinical pathology, anatomical pathology	Clinical reactions, bodyweight, food and water consumption, clinical pathology, anatomical pathology	Clinical reactions, bodyweight, food and water consumption, clinical pathology
Age of cats at study start	8–10 weeks	8–9 weeks	7–9 weeks

1× Group: 4.5 mg/kg esafoxolaner, 1.5 mg/kg eprinomectin, 31.1 mg/kg praziquantel (maximum exposure dose: 0.375 mL/kg).

3× Group: 13.5 mg/kg esafoxolaner, 4.5 mg/kg eprinomectin, 93.4 mg/kg praziquantel (3X maximum exposure dose: 1.125 mL/kg).

5× Group: 22.5 mg/kg of esafoxolaner, 7.5 mg/kg eprinomectin, 155.6 mg/kg praziquantel (5X maximum exposure dose: 1.875 mL/kg).

Twenty-one healthy Domestic Short-hair kittens (11 males and 10 females) were studied. The acclimation of all kittens to study conditions started on Day-7. On Day −1, all 21 kittens were weighed (0.8–1.4 kg). One male and one female were randomly allocated to the control group. The 19 remaining kittens were ranked by decreasing body weight and formed six blocks of three kittens each, plus one last kitten. Within each block, the kittens were randomly allocated to one of the three treatment groups, and the 19th lightest (female) kitten was allocated to the 5× group. Consequently, two kittens were assigned to the control group, six to the 3× combination product group, seven to the 5× combination product group, and six to the esafoxolaner group. The kittens were 8–10 weeks of age on Day 0.

Animals from the 3× and 5× combination product groups were treated on Days 0, 14, 28, and 42, animals from the esafoxolaner group were treated on Days 0 and 28. Treatments were administered topically, by parting the hair in one spot, and were applied directly on the skin on the midline of the neck between the base of the skull and the shoulder blades, with a precision of 0.01 mL (to avoid run-off, the product was administered in fractions, on the same spot).

Clinical observations were performed at least once a day: on dosing days, prior to, then 1–4, 6 and 8 hours after treatment; twice a day the two days following each treatment; otherwise once a day. The clinical observations included assessment of general health, scoring of pupillary dilation and light reflex, and observation of the cage for any abnormality (e.g. diarrhea, body fluid). Bodyweights were measured weekly from Day −1 to Day 57.

Blood and urine samples were collected at baseline and on Days 29 and 57 for clinical pathology parameters including hematology, serum biochemistry, coagulation factors, and urinalysis, as listed in Table 2.

**Table 2:** Clinical pathology and histology parameters.

	Preliminary margin-of-safety topical (Study #1)	Regulatory margin-of-safety topical (Study #2)	Regulatory oral tolerance (Study #3)
Hematology			
RBC (red blood cell count)	√	√	√
WBC (white blood cell count, absolute and differential)	√	√	√
HGB (hemoglobin concentration)	√	√	√
HCT (hematocrit)	√	√	√
Platelet count	√	√	√
MCV (mean corpuscular volume)	√	√	√
MCH (mean corpuscular hemoglobin)	√	√	√
MCHC (mean corpuscular hemoglobin concentration)	√	√	√
Reticulocytes (% and absolute)		√	
LUC (large unstained cell)		√	
RDW (red cell distribution width)		√	
Platelet estimate		√	
RBC morphology		√	
Serum biochemistry			
Total Protein	√	√	√
Albumin	√	√	√
Globulin (calculated)	√	√	√
Total bilirubin	√	√	√
Blood urea nitrogen	√	√	√
Creatinine	√	√	√
ALT (alanine aminotransferase)	√	√	√
ALP (alkaline phosphatase	√	√	√
AST (aspartate aminotransferase)	√	√	√
GGT (gamma glutamyl transferase)	√	√	
Glucose	√	√	√
Total cholesterol	√	√	√
Calcium	√	√	√
Chloride	√	√	√
Phosphorus	√	√	√
Potassium	√	√	√
Sodium	√	√	√
Triglycerides	√	√	√
Magnesium		√	
Appearance (e.g. degree of hemolysis, lipemia, icterus)		√	
Coagulation factors			
PT (prothrombin time)	√	√	
Fibrinogen	√	√	
APTT (activated partial thromboplastin time)	√	√	
Urinalysis			
SG (specific gravity)	√	√	√
pH	√	√	√
URO (urobilinogen)	√	√	√
COL (color)	√	√	
CLA (clarity)	√	√	
PRO (protein)	√	√	√
GLU (glucose)	√	√	√
KET (ketones)	√	√	√
BIL (bilirubin)	√	√	√
Nitrite			√
BLD (occult blood)		√	√
Microscopy of sediments (crystals, RBC, WBC, epithelial cells)	√	√	√
Histology			
Gross lesions (when applicable)	√	√	
Heart	√	√	
Kidneys (2)	√	√	
Liver (sections of two lobes)	√	√	
Spleen	√	√	
Thymus	√	√	
Lungs (including bronchi)	√	√	
Skin from application site	√	√	
Skin from abdomen	√		
Skin with mammary gland		√	
Adrenals (2), aorta, bone with marrow, femur, sternum, bone marrow smear, brain, cervix, eyes with optic nerves (2), gallbladder, esophagus, stomach, duodenum, jejunum, ileum, cecum, colon, rectum, lymph nodes (mandibular, mesenteric, prescapular (2)), ovaries with oviducts (2), pancreas, sciatic nerve, peyer’s patches, pituitary, prostate, mandibular salivary gland (2), skeletal muscle (femur rectoris), spinal cord (cervical, thoracic, lumbar), testes with epididymis (2), thyroids with parathyroids (2), tongue, trachea, ureters, urinary bladder, uterus, vagina		√	

All cats were necropsied on Day 57, starting with an external examination of the body, of all orifices, and followed by examination of the oral, nasal, cranial, thoracic, abdominal, and pelvic cavities, including viscera. Specific tissues (as listed in Table 2) were collected and processed for histology and histopathological examination by a board-certified pathologist. Kidneys and liver with drained gall bladder were weighed for all animals.

### Study #2 regulatory margin of safety

The design of Study #2 is summarized in Table 1. Thirty-two healthy Domestic Short-hair kittens (16 males and 16 females) were included in this blinded margin-of-safety study. The acclimation of all kittens to study conditions started on Day −14. The cats were 8–9 weeks of age on Day 0 and weighed 0.7–1.2 kg on Day −1.

On Day −2, the 32 kittens were weighed and ranked by decreasing body weight within sex. For each sex, 4 weight blocks of 4 kittens were created for random allocation to the four treatment groups; a control group treated with a placebo (mineral oil), and three groups treated with NexGard^®^ Combo at 1×, 3× or 5× the maximum exposure dose. All animals were administered six consecutive doses monthly, on Days 0, 28, 56, 84, 112, and 140.

Treatments were administered topically, by parting the hair in one spot, and were applied directly on the skin on the midline of the neck between the base of the skull and the shoulder blades, with a precision of 0.01 mL (to avoid run-off, the product was administered in fractions on the same spot, for the 3× and 5× dose groups).

Throughout the study, beginning on the first day of acclimation and until the end of the in-live phase, clinical observations were performed twice daily. On dosing days, clinical observations were performed prior to dosing, then 10 min and 1–4, 6 and 8 hours after treatment, and also 7, 9 and 10 h after treatment from Day 84. These clinical observations included assessment of general health, scoring of pupillary dilation and light reflex, and observation of the cage for any abnormality (e.g. diarrhea, body fluid).

Detailed physical examinations were performed on Days −14, −5, −1, weekly during the study period, on the day following each treatment, and on the day of necropsy. These examinations included measurement of rectal temperature, respiration rate, heart rate, bodyweight, examination of the head including oral cavity and eyes (e.g. nystagmus, congestion, discharge, signs of visual impairment, pupil size and responses), skin/haircoat, lymph nodes, respiratory system and circulatory system (including chest auscultation), digestive system including abdominal palpation, musculoskeletal system, autonomic and central nervous system, somato-motor activity, genitalia, and dosing application site.

Food and water consumption were measured daily throughout the study starting on Day −1.

Blood and urine samples were collected at baseline and on Days 29, 57, 85, 113, 141, and 168 or 169 for clinical pathology parameters including hematology, serum biochemistry and urinalysis, as listed in Table 2.

All cats were necropsied on Days 168 or 169, starting with an external examination of the body, of all orifices, and of the oral, nasal, cranial, thoracic, abdominal, and pelvic cavities, including viscera. Specific tissues and lesions (as listed in Table 2) were collected and processed for histology and histopathological examination by a board-certified pathologist. A formal pathology peer-review was conducted by a second board-certified pathologist. The organs (brain, heart, paired weight of kidneys, liver with drained gall bladder, paired weight of ovaries with oviducts, and paired weight of testes with epididymis) were weighed for all animals. Organ to body weight ratio (using the terminal body weight) and organ to brain weight ratios were calculated.

### Study #3 regulatory oral tolerance

The design of Study #3 is summarized in Table 1. Sixteen healthy kittens, 8 females and 8 males, aged 7–9 weeks on Day 0, and weighing 0.6–1.0 kg were studied in this blinded oral safety study. The acclimation of all kittens to study conditions started on Day-7. Four females and 4 males were randomly allocated to the control group, or to the NexGard^®^ Combo group.

All kittens were treated once on Day 0, either with sterile water or with the combination product, at 1× the maximum exposure dose (0.375 mL/kg) administered directly in the mouth using a syringe aiming at the back of the tongue. Clinical observations were performed twice daily, from the first day of acclimation, until the end of the in-live phase on Day 14. On Day 0, clinical observations were performed 1–4, and 8 hours following treatment. Detailed physical examinations were performed on Day −4, Day 0 (6 h post-treatment), Days 1, 3, 7 and 14. Clinical observations and detailed physical examinations were conducted the same way as in Study #2. Blood and urine samples were collected on Days −1 and 14, for clinical pathology analyses (hematology, clinical biochemistry and urinalysis, as listed in Table 2). Daily feed intake was measured for each animal during the entire in-life phase.

## Statistical analysis

**Study #1** Group means and individual values of the clinical pathology parameters (hematology, plasma chemistry, coagulation and urinalysis) were compared with the baseline values instead of the concurrent controls due to small animal number in the control group (*n* = 2). No statistical analysis was performed in this study.

**Study #2** clinical pathology parameters (biochemistry, hematology values, and urine specific gravity only), respiration rate, temperature, heart rate, and body weight were analyzed using repeated measures analysis of covariance (RMANCOVA) including treatment, sampling day, sex, and the interaction terms “treatment by sex,” “treatment by sampling day,” “sex by sampling day,” and “treatment by sex by sampling day” as fixed effects. Allocation block-within-sex group was included as the random effect. Sampling day was the repeated fixed effect and “block by treatment within sex” was identified as the subject in the repeated statement. The covariate was the most recent measurement prior to the start of treatment. The variables food and water intake were analyzed using repeated measures analysis using the same model as was used with RMANCOVA but without the baseline covariate (RMANOVA). Organ weights were analyzed using analysis of variance (ANOVA) including treatment, sex, and the interaction term “treatment by sex” as fixed effects. Allocation block-within-sex group was included as the random effect. The organ weights were analyzed both absolute, as well as relative to body weight and relative to brain weight.

**Study #3** clinical pathology parameters (biochemistry, hematology values, and urine specific gravity only) were analyzed using analysis of covariance (ANCOVA), and following paradigm was followed. The interaction of “treatment by sex” was evaluated at the 0.10 significance level. If this interaction was significant, then the treatment group was compared with the control group within sex at the 0.10 level of significance. If “treatment by sex” interaction was not significant, then at the main effect level, the treatment group was compared with the control group at the 0.10 level of significance without blocking for sex. The covariate was the pre-treatment measurement taken on Day −1. Age matched kitten clinical pathology reference intervals were also available. Feed consumption was analyzed using ANCOVA. The covariate was the pre-treatment measurement. Feed consumption for each animal was combined into three summaries: pre-treatment days; Days 0 through 7; and Days 8 through 14. For each summary, the daily average weight of feed consumption was estimated using descriptive statistics.

## Results

### Study #1

Histologically, one animal each in the 5× combined formulation and the esafoxolaner groups had a non-adverse acute, focal epidermal erosion of minimal severity. A small number of transient and self-limiting mild to moderate pupil dilations of little clinical significance and with normal response to light were observed in the 3×, 5× combined formulation, and the 23× esafoxolaner groups, mostly during the 6 h following the Day 0 treatment.

### Study #2

No treatment related effects were observed on body weight, food consumption, water consumption, clinical pathology parameters (hematology, coagulation, serum biochemistry, and urinalysis), organ weights, and histopathological observations at any dose level. At necropsy, three cats in the 5× group exhibited single small dark red subcutaneous area at the treatment sites. No histopathological abnormalities were observed correlating these macroscopic observations upon the microscopic examination of these sites. Considering the low severity degree of the macroscopic changes, the absence of related clinical signs and in the absence of histological abnormalities, this observation is considered non-adverse and not critical for the outcome of the tolerance of the product at 5× the maximum dose.

One reversible adverse event was observed in a 5× male cat after administration of the third dose on Day 56. Before the third treatment, and during the 6-h post-dosing period, the cat appeared normal except for the presence of soft feces in the cage. Nine hours after treatment, the cat had an increased respiration rate and was hypoactive although responsive to stimuli, with disorientation, dorsal recumbency with ataxia and inability to stand, slight tremors, hypothermia 35.1 °C (95.1 °F), and dilated pupils with slight pupillary light reflex. Two hours later, the application site was washed off and diazepam was administered along with sub-cutaneous fluids. The next morning, the cat was first described as bright, alert, playful and mildly ataxic with normal pupils and body temperature. Then another observer noted decreased activity and dilated pupils responsive to light. The scheduled blood collection was performed and the results were normal. The next day (two days post dosing), the cat was normal except for injected sclera. This cat had no adverse reaction after the next three 5× dosings (Days 84, 112, and 140) and it had a normal growth rate and no abnormal clinical or anatomical pathology observation throughout the study.

### Study #3

Profuse salivation was observed immediately after the treatment in all 1× NexGard^®^ Combo-treated kittens. One hour after treatment, the mouth of all these kittens appeared normal with no excessive salivation.

No other clinical adverse reactions were observed throughout the study. No significant difference was observed for food consumption among the treated and control groups. No statistically significant and clinically meaningful changes were observed in any of the hematology, plasma biochemistry, and urinalysis parameters in males or females in the treated and the control groups.

## Discussion

The safety of eprinomectin and praziquantel is well established. Eprinomectin has been used in ruminants for more than two decades in pour-on formulations [14]. Praziquantel has been the platyhelminth treatment of choice in companion animals as well as in humans over several decades [3, 6, 7]. Praziquantel is provided in the novel formulation at doses comparable to existing topical products for cats (Droncit^®^ Spot-on, Profender^®^ Spot-on). Eprinomectin and praziquantel are present at the same recommended doses as in Broadline^®^ and Centragard^®^, two registered products for the treatment, control and prevention of a broad spectrum of feline parasites [5].

The safety of Broadline^®^ a combination of fipronil, (S)-methoprene, eprinomectin, and praziquantel, was established during the development of this product and included three regulatory target animal safety studies: a margin-of-safety study in kittens, a safety study in heartworm-positive cats, and an oral tolerance study in cats. The Broadline^®^ margin-of-safety study was conducted using an identical study design, and similar animals and inclusion criteria as those employed in Study #2. In the Broadline^®^ study, one kitten treated at 5× the maximum dose exhibited an adverse reaction comparable to the one described in Study #2: ataxia, disorientation, and lethargy for 12 h and pupil dilation for 24 h following the third treatment. That kitten exhibited ataxia, disorientation, lethargy, and moderate pupil dilation following the fourth treatment; and showed no abnormality after the following subsequent fifth and sixth treatments.

Broadline^®^ was also tested in an oral tolerance study using an identical design to that employed in Study #3, except for the age of the cats, which were 9–10 months old. Similarly to the study described in this manuscript, the cats dosed orally with Broadline^®^ 1X exhibited hypersalivation immediately and for two hours after oral administration. Two cats also vomited and three cats were temporarily lethargic after the oral Broadline^®^ administration, but no other relevant clinical observations was reported from 2 h after dosing and over the following 14 days.

The adverse reaction observed in the 5× novel formulation-treated male cat dosed with 5× of the novel formulation in the margin-of-safety Study #2 reported here was associated with the administration of the third dose of the novel combination on Day 56. The clinical signs of ataxia, disorientation, and pupillary dilation were similar to the clinical signs reported in one 5×-dosed kitten in the Broadline^®^ margin-of-safety study (Sponsor’s unpublished data), and may be attributed to eprinomectin since they reflect signs associated with macrocyclic lactone toxicity. In dogs and cats, signs of macrocyclic lactone toxicity may include neurological depression, ataxia, pupil dilation, temporary sight impairment, tremors and hypersalivation [11]. The cat in Study #2 showed improvement overnight, and was normal on the following day and at subsequent examinations. This cat showed no abnormal signs following the next three 5× dosings, had a normal growth rate and no significant clinical, gross or microscopic finding at the scheduled necropsy examination at the end of the study.

The transient hypersalivation signs seen in the 1× novel formulation treated cats in Study #3 was expected following oral administration of this novel formulation. These signs are attributed to the bitter taste of praziquantel [12, 13] rather than any pharmacological reaction due to active ingredients in the investigated product.

In the preliminary margin of safety study, a very high dosage of topical esafoxolaner was also investigated, as this is a novel compound both in veterinary medicine and in felines. Esafoxolaner was administered two times at a 4-week interval at 103.1 mg/kg, a dosage corresponding to 23× the maximum exposure dose of NexGard^®^ Combo (i.e. 4.5 mg/kg). An excellent tolerance to very high overdosage of topical esafoxolaner was demonstrated in cats, as only a few incidences of pupil dilations and a histological reaction at application site, both with little clinical significance were observed.

The safety of the racemic form of afoxolaner, which includes esafoxolaner, has been well demonstrated in dogs [4]. Afoxolaner is an active ingredient approved for the treatment and control of arthropods in dogs. Afoxolaner was well tolerated when administered orally to 8-week-old Beagle dogs at 1, 3, or 5× the maximum potential exposure dose of 6.3 mg/kg at three, 28-day dose intervals followed by three, 2-week dose intervals for a total of 6 doses.

Results presented here confirm the safety of NexGard^®^ Combo, including the high safety margin of esafoxolaner in kittens.

Additionally, the safety of NexGard^®^ Combo was confirmed in parasite-infested cats during the development of the product, in multiple studies using experimental or natural infestation models, where no significant adverse reactions related to treatment were observed. For example, in the flea field trials, more than 700 cats were treated and assessed for safety worldwide [17]. Most of these cats were infested with fleas and a significant number of them were likely also infested with endoparasites because poly-parasitism is frequent in cats in the field, as shown in a multicentric study where 35.1% of 1519 domestic and healthy cats were infected with endoparasites [2].

## Conclusions

NexGard^®^ Combo was well tolerated and demonstrated safe for domestic cats when administered topically at fortnightly or monthly intervals. Additionally, it was well tolerated when administered once orally to kittens at the maximum exposure dose, and two topical applications at a 4-week interval of a solution of esafoxolaner dosed at 23× the maximum exposure dose of NexGard^®^ Combo were well tolerated.

## Competing interest

The work reported herein was funded by Boehringer-Ingelheim. The authors are current employees of Boehringer-Ingelheim. Other than that, the authors declare no conflict of interest. This document is provided for scientific purposes only. Any reference to a brand or trademark herein is for information purposes only and is not intended for any commercial purposes or to dilute the rights of the respective owners of the brand(s) or trademark(s).

NexGard^®^ is a registered trademark of the Boehringer-Ingelheim Group.
